# Universal Health Coverage through Community Nursing Services: China vs. Hong Kong

**DOI:** 10.1590/1518-8345.1664.2838

**Published:** 2017-01-30

**Authors:** Wai Yee Chan, Ita M Fung, Eric Chan

**Affiliations:** 1 DHSc, Assistant Professor, School of Health Sciences, Caritas Institute of Higher Education, Hong Kong, China.; 2 PharmD, Associate Professor, School of Health Sciences, Caritas Institute of Higher Education, Hong Kong, China.; 3 DMgt, Professor, School of Health Sciences, Caritas Institute of Higher Education, Hong Kong, China.

**Keywords:** Public Health, Community Health Nursing, Global Health, Hong Kong

## Abstract

**Objective:**

this article looks at how the development of community nursing services in China and Hong Kong can enhance universal health coverage.

**Methods:**

literature and data review have been utilized in this study.

**Results:**

nursing services have evolved much since the beginning of the nursing profession. The development of community nursing services has expanded the scope of nursing services to those in need of, not just hospital-level nursing care, but more holistic care to improve health and quality of life.

**Conclusion:**

despite the one-country-two-systems governance and the difference in population and geography, Hong Kong and China both face the aging population and its complications. Community nursing services help to pave the road to Universal Health Coverage.

## Introduction

The WHO Constitution of 1948 declared that *health* is a fundamental human right, which is defined as a state of physical, mental and social wellbeing, and is not just the absence of disease or infirmity^1^ ^-^ ^2^. Upholding the declaration in the WHO Constitution of 1948, The Alma-Ata Declaration of 1978 identified primary health care as care to “address main health problems in the community, [and provide] preventive, curative and rehabilitative services”^2^. As time elapses, the term, Universal Health Coverage, came about to ensure all people can receive the necessary promotive, preventive, curative, rehabilitative and palliative health services of quality without financial difficulties imposed on the users in terms of service payment^3^ . With the aim to improve health through the delivery of quality and affordable health services to all people, the governments have an inescapable role. 

Although Hong Kong is part of China, the governing mechanisms in these two places are different for historical reasons. The provision of health services to their citizens relies greatly on the public sector in both places. The citizens in both places tend to seek medical attention or health services from the public tertiary medical institutions - hospitals^4^. Despite the implementation of various measures to “mobilize” the citizens to the “non-hospital” settings for basic health services in both China and Hong Kong, the health seeking behavior, for a more subjectively reliable and/or cheaper medical services, of their citizens seems to be negatively impacting on the effectiveness of these measures^4^. With public health sector still being the more frequently used health service provider, this article looks at how the Hong Kong government and the Chinese government enhance universal health coverage through community nursing services.

## Community Nursing Service in Hong Kong

The population aged 65 or above will double in Year 2041 and the numbers of disability-adjusted life years (DALYs) attributed to the age-dependent chronic diseases is projected to increase vastly by Year 2030(5). In 2014, Prince et al. forecasted the DALYs of ischemic heart diseases, cerebrovascular disease, diabetes mellitus, and dementia will increase by 34.7%, 44.4%, 95.7%, and 82.6%, respectively^5^. These statistical projections are surely a good forecast to the challenges facing the health care providers. 

The development of community nursing services in Hong Kong started in 1967^6^. The services carry four purposes^6^:

- providing individualized and continuing nursing care for patients at home;
- maximizing patients’ self-care ability and providing resolution to home care problems;
- decreasing the number and duration of hospitalizations; and
- improving patients’ quality of life.

Today, Community Nursing Services provided by the public sector in Hong Kong consist of 1. community psychiatric nursing service, which provides nursing services to the mental patients living in the community^7^; the community psychiatric nurses also provide crisis intervention service^6^ ^-^ ^7^ and report to the doctors on the patient’s progress^7^; 2. community nursing services, of which the service focuses on providing general nursing care, special nursing care, health education, and home rehabilitation services^8^; 3. community geriatric assessment team, which visit the residential care homes for the elderly to provide multi-disciplinary services and community-based rehabilitation programs to the residents^9^; and 4. community psychogeriatric team, which provides designated care and rehabilitation programs to the patients^10^.

Another challenge arising from the increase in the ageing population is the increase in demand for post-hospitalization nursing care, especially for the frail elderly, as it had been reported by the New South Wales Department of Health that attendance at the emergency department and unplanned hospital readmissions are common for frail elderly after being discharged from the hospital^11^. Moreover, the anticipatory increase in age-dependent chronic diseases also poses a demand for post-hospitalization nursing care as Yu et al. mentioned that upon discharge, patients with chronic diseases tend to seek hospital readmission immediately upon symptom exacerbation^11^. To enhance the quality of community nursing care, the health care model of “virtual ward” has been brought into the light. This model was implemented in October 2011 in Hong Kong^12^. Aiming at reducing avoidable hospitalization and improving the quality of life, “virtual ward” is a hospital-level care at patient’s home offering comprehensive multidisciplinary care coordinated and led by nurses^12^. A pilot study on the effect of the virtual ward, published in 2015, concluded with a reduction in unplanned hospital readmission and quality of life improvement^11^. 

## Community Nursing Service in China

Community health nursing (CHN) has been recognized as a long-term development strategy in China’s health care system when the Ministry of Health of China pointed it in the “Notice on Strengthening Nursing Management (File No. 23)” in 1997^13^ ^-^ ^14^. 

CHN is delivered by two main organizations, which the financial support of both of them comes from central and local governments, including community health service (CHS) centers and their sub-centers. The structure of CHS centers and sub-centers is simple that three to six sub-centers attached to one CHS center. Sub-centers report cases to CHS center that CHS centers report cases to the local health department. Each sub-center services about 10,000 populations, and each CHS center services about 30,000-100,000 populations^13^ ^,^ ^15^. As supported by central government, the numbers of CHS centers and sub-centers increased rapidly that CHS centers showed a 400% increase and sub-centers showed a 64% increase by the end of 2010. More than 95% of cities in China have CHS centers and sub-centers^13^ ^,^ ^16^ ^-^ ^17^. 

CHS centers and sub-centers have similar functions, including medical care, disease prevention, health care, rehabilitation, health education, and birth control^13^. 

But some daily works duplicate with hospital care, such as intravenous infusions, injections, and dressing. The nursing staff spend a little time in disease prevention and health education^13^ ^,^ ^18^ ^-^ ^19^.

## Enhancement of Universal Health Coverage through Community Nursing Services

### Hong Kong

To enhance Universal Health Coverage, the typical community nursing services in Hong Kong have expanded the service scope to the patient’s residence. This improves the accessibility of health services to those in need in the community by broadening the geographic coverage by the nursing services. The quality of care is also enhanced because the nurses’ professional knowledge and judgment ensure that the patients are properly assessed, served, and most importantly, referred to the other health care professionals for appropriate care. In addition, stepping into a patient’s residence offers a more comprehensive picture of the patient’s health condition for better clinical decision-making, and thus, facilitating more holistic and proper care. 

By implementing the model of “virtual ward”, the scope of community nursing services is widened. The nursing care provided serves as an interface for acute institutional care and “upgraded” home care. The need to enhance the skills of community nurses is now called upon.

### China

Although some of the work of CHN duplicate with that of hospital care in China, the service of CHN is improving health care accessibility in China; and the scope of CHN service places much focus on promoting public health. These are an effort made to moving its way forward to universal health coverage. The Central government of China established CHS centers and sub-centers in place in every neighborhood within a 15-minute walking distance to ensure close-to-home primary care^20^ ^-^ ^21^. The government provides financial support on the basic public health (BPH) service package, which is determined by the number of citizens using CHN centers’ services. The BPH services are broad and comprehensive, including (a) to establish health profiles and medical records; (b) to provide health education; (c) disease prevention and vaccination; (d) to provide health management for the elderly, pregnant women, children and citizens with hypertension, diabetes or serious mental illness; (e) to control infectious diseases and public health emergencies and (f) to monitor public health^21^ ^-23)^. 

Comparing to Hong Kong, China’s community health nursing services are still center based; patients need to be physically present in the CHN center to receive services. To further enhance the effectiveness of CHN in china, organizational change in the service delivery model and enhanced scope of nursing practice will be needed. While service model providing enhanced nursing services in the community, such as the “virtual ward” model, is still quite distant and geographically challenging for a big country, like China. 

## Conclusion

The nature, focus, and model of delivery of community nursing services differ between Hong Kong and China. In Hong Kong, community nursing services are provided at patients’ home aiming at caring for patients’ active condition and improving patients’ quality of life. The provision of more in-depth services and enhancing the nursing service quality are important. Training and enhancement in advanced nursing skills play an important role. In China, community nursing services are provided in community health centers focusing on improving public health. Geographic differences, population, and population distribution are factors for consideration in determining the services to be delivered and the mode of delivery. More “into-the-community” services, such as a “virtual ward” for less complicated patient cases, can further enhance service accessibility, improve the quality of life, and extend the service to those who have difficulty accessing the CHN centers for services. This kind of service also calls upon the collaboration of various health professionals. Trying out these service models in major cities may be an initial step and a positive result may help further enhance Universal Health Coverage in such a big country.

Community-based nursing services present a great opportunity for nurses to enhance their contributions to Universal Health Coverage. It is certain that community nursing in Hong Kong will continue to evolve for better and more specific contribution to those in need in the community. Whilst similar potential does exist in China, nurses will have to advocate for more “targeted” service model to enhance care coverage, as well as expanding the scope and depth of community nursing services.
